# Association of Psychiatric History and Type D Personality with Symptoms of Anxiety, Depression, and Health Status Prior to ICD Implantation

**DOI:** 10.1007/s12529-012-9244-3

**Published:** 2012-07-22

**Authors:** Annemieke H. Starrenburg, Karin Kraaier, Susanne S. Pedersen, Moniek van Hout, Marcoen Scholten, Job van der Palen

**Affiliations:** 1 Department of Clinical Psychology, Medisch Spectrum Twente, P.O. Box 50.000, 7500 KA Enschede, The Netherlands; 2 Department of Cardiology, Thorax Center Twente, Medisch Spectrum Twente, Enschede, The Netherlands; 3 CoRPS-Center of Research on Psychology in Somatic Diseases, Tilburg University, Tilburg, The Netherlands; 4 Department of Research Methodology, Measurement and Data Analysis, University of Twente, Enschede, The Netherlands

**Keywords:** Anxiety, Depression, Health status, ICD, Type D personality

## Abstract

**Background:**

Personality factors and psychiatric history may help explain individual differences in risk of psychological morbidity and poor health outcomes in patients with an implantable cardioverter defibrillator (ICD).

**Purpose:**

We examined associations between previous anxiety and depressive disorder, type D personality, anxiety or depressive symptoms, and health status in ICD patients prior to ICD implantation.

**Method:**

Patients (*N* = 278; 83 % men; mean age = 62.2 years ±11) receiving a first ICD from September 2007 through April 2010 at the Medisch Spectrum Twente, The Netherlands completed validated questionnaires before implantation assessing type D personality (14-item Type D Scale), anxiety and depressive symptoms (Hospital Anxiety and Depression Scale), and health status (36-item Short Form Health Survey). History of anxiety or depressive disorder was assessed with the Mini International Neuropsychiatric Interview structural interview.

**Results:**

Previous anxiety or depressive disorder was prevalent in 8 and 19 % of patients, respectively. Type D personality was present in 21 %, depressive symptoms in 15 %, and anxiety in 24 %. In adjusted analyses, type D personality was a dominant correlate of previous depressive disorder (odds ratio (OR) 6.2, *p* < 0.001) and previous anxiety disorder (OR 3.9, *p* = 0.004). Type D personality (OR 4.0, *p* < 0.001), age (OR 1.03, *p* = 0.043), and gender (OR 2.5, *p* = 0.013) were associated with anxiety symptoms at baseline. Type D personality (OR 5.9. *p* < 0.001) was also associated with increased depressive symptoms at baseline. Heart failure and type D personality were related to poorer health status.

**Conclusion:**

In ICD patients, prior to ICD implantation, a previous anxiety or depressive disorder, type D personality, and anxiety and depressive symptoms were associated with poorer health status. Type D personality was also independently associated with increased anxiety and depression symptoms.

## Introduction

Sudden cardiac death (SCD) is a common manifestation of coronary heart disease (CHD) and is responsible for about 50 % of deaths from cardiovascular disease in developed countries [1]. Several primary and secondary prevention trials have demonstrated the efficacy of the implantable cardioverter defibrillator (ICD) compared to anti-arrhythmic drugs in reducing the number of SCDs by treating potentially life-threatening ventricular arrhythmias [2–4]. ICDs prevent SCD by converting ventricular arrhythmias into a normal rhythm by the use of anti-tachycardia pacing or shock therapy. A subset of ICD patients have difficulty with psychological functioning following implantation, with 25–33 % of patients displaying significant symptoms of anxiety, depression, and posttraumatic stress [5–8]. In turn, preimplantation psychological morbidity, such as anxiety, depression, and posttraumatic stress, have been shown to negatively influence mortality, morbidity, and health status in ICD patients [7, 9–11].

The manifestation of psychological morbidity may be attributed to a combination of factors, including the ICD implantation, the occurrence of ICD shocks, the type of underlying heart disease and psychological symptoms, such as anxiety and depression [12, 13]. Preliminary evidence suggests that personality factors may also help to identify patients at risk for developing adverse health outcomes in the future [14–18]. Type D personality, defined as the tendency to experience negative emotions combined with the tendency to inhibit these emotions in social interactions [14], seems to constitute a vulnerability factor for future anxiety, depression [19], and poor health status [16], regardless of the underlying cardiac disease pathology. Moreover, anxious ICD patients with a type D personality have a 70 % increased risk of ventricular arrhythmias, possibly due to the modulating effect of personality on emotional distress [20] and also a higher risk of mortality [21].

Most psychological studies in ICD recipients have focused on symptoms after implantation, neglecting the role of a previous history of psychiatric disorders [10, 22, 23]. To our knowledge, this has only been investigated in one study, showing that a previous diagnosis of depression predicts poor health status in ICD patients [18]. This despite findings demonstrating that a previous history of psychiatric disorder has a significant impact on the development of heart disease [24]. After myocardial infarction, a history of anxiety disorder predicts the occurrence of reduced heart rate variability [25], a known risk factor for future ventricular arrhythmia.

In ICD patients, it has been shown that distress (as reflected by increased anxiety and depressive symptoms) and poor health status at the time of implantation are risk factors for the onset of ventricular arrhythmias but also for mortality [10, 20, 21, 26]. Increasing our understanding of which personality factors are associated with an increased risk of psychological distress prior to ICD implantation may help health care professionals to identify patients at risk in order to optimize their clinical management and care. The objective of the current cross-sectional study was to examine the association between type D personality and a previous anxiety or depressive *disorder*, and current health status, but also the association between type D personality and anxiety- or depressive *symptoms* prior to ICD implantation.

## Method

### Patients and Design

**Fig. 1:**
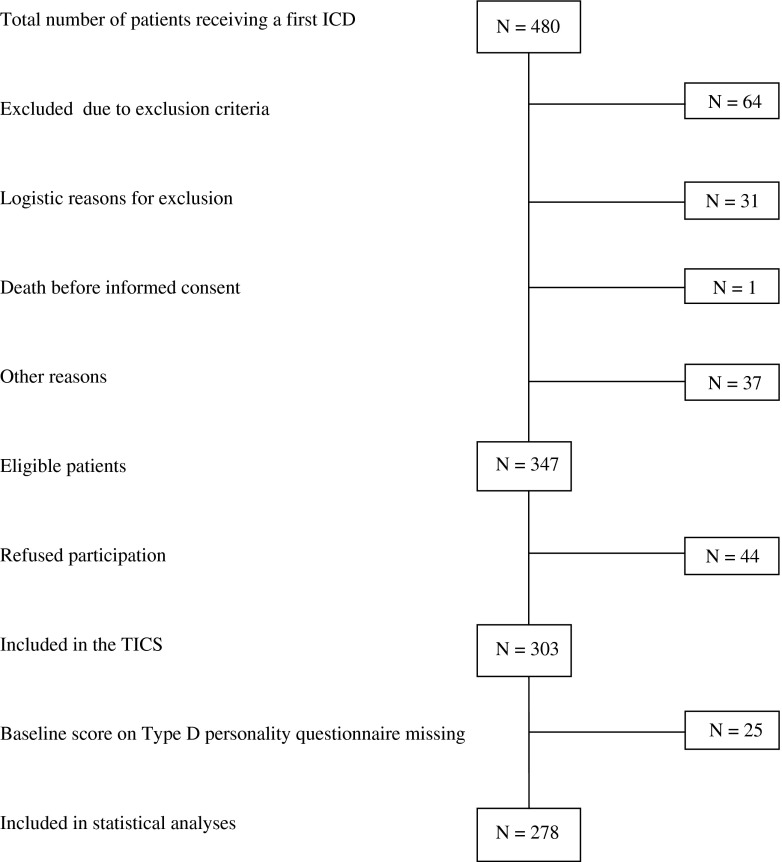
Patient selection for the current study

The study population consisted of a consecutive cohort of patients implanted with an ICD at Medisch Spectrum Twente (*N* = 278; 83.4 % men; mean age = 62.2 years ±11), Enschede, The Netherlands, between September 2007 and April 2010, as part of the Twente ICD Cohort Study (TICS, American trial register number NL13939.044.06). The TICS study was designed to examine the value of biomedical and psychological risk markers for predicting life-threatening ventricular arrhythmias requiring ICD therapy. Patients meeting the following inclusion criteria were eligible for participation in the study: (1) an indication for a first ICD implantation according to the guidelines of The Netherlands Society of Cardiology and the European Society of Cardiology [2, 27, 28], (2) age ≥18 years at the time of implantation, and (3) providing written informed consent. Exclusion criteria were inability to read or write Dutch, congenital heart disease, major psychiatric disorders other than affective spectrum disorders (e.g., psychosis, dementia), and participation in other studies. The included patients completed a set of standardized and validated questionnaires before ICD implantation, referred to as baseline in the remainder of the paper. Twenty-five patients did not return the questionnaires. With a response rate of 80.1 %, statistical analyses were based on 278 patients (Fig. 1). The study was conducted according to the Helsinki Declaration and the research protocol was approved by the local ethics committee. All patients provided written informed consent.

### Measures

#### Demographic and Clinical Variables

Information on demographic and clinical variables was collected from the patients’ medical records before implantation. Clinical variables included indication for ICD placement (i.e., primary versus secondary prevention), etiology (i.e., ischemic versus non-ischemic), comorbidity (i.e., chronic obstructive pulmonary disease, hypertension, or diabetes mellitus), smoking, alcohol use, New York Heart Association (NYHA) functional class, left ventricular ejection fraction (LVEF), and medication (i.e., beta blockers, amiodarone, diuretics, aldosterone antagonist, and ACE inhibitors).

#### Psychological Questionnaires

##### Anxiety and Depressive Symptoms

Symptoms of anxiety and depression were evaluated with the Hospital Anxiety and Depression Scale (HADS) [29]. The HADS is a validated self-report measure consisting of 14 questions that tap into current symptoms of anxiety and depression. Items are answered on a four-point Likert scale (0–3), with a score range of 0–21 for both anxiety and depression. A higher score indicates more symptoms of anxiety or depression. The HADS was designed to be used as a screening instrument and not as a case identifier for anxiety or depressive disorder [30]. Scores on both scales ≥8 are indicative of elevated anxiety or depressive symptoms [31]. The HADS has sufficient to good psychometric properties, as indicated by Cronbach’s *α* = 0.71 and *α* = 0.90 for the anxiety and depression subscales, respectively, and test–retest reliability of 0.86 and 0.91, respectively [30].

##### History of Depressive or Anxiety Disorder

History of anxiety disorder or depressive disorder was measured with the Dutch version of the Mini International Neuropsychiatric Interview (MINI) [32, 33]. This is a structured diagnostic interview that systematically determines psychiatric diagnoses. It was developed to meet the need for a short but accurate structured psychiatric interview for multicenter clinical trials and epidemiological studies, addressing the feasibility shortcomings of two other frequently used structured psychiatric interviews, the Structured Clinical Interview for DSM-IV-TR Axis I and the Composite International Diagnostic Interview. In this study, only the parts relating to depressive disorder and anxiety disorder were administered. Test–retest reliability Kappa scores are 0.89 and 0.79, inter-rater Kappa scores are 1.00 and 0.97, respectively [32].

##### Type D Personality

Type D personality was measured with the 14-item Type D Scale (DS14) [34], a self-report questionnaire assessing the personality traits negative affectivity (seven items; e.g., “I often feel unhappy”) and social inhibition (seven items; e.g., “I am a closed kind of person”). Items are answered on a five-point Likert scale from 0 (not true) to 4 (true), with scores ranging from 0 to 28 for both subscales. Type D caseness is defined by a score of ≥10 on both subscales [34], with this cutoff shown to be the most optimal according to item response theory [35]. The DS14 has good psychometric properties with Cronbach’s *α* = 0.88/0.86 and 3-month test–retest reliability = 0.72/0.82 for the negative affectivity and the social inhibition subscales, respectively [34]. Type D personality is not confounded by indicators of disease severity, such as left ventricular dysfunction, in post myocardial infarction patients [36, 37], nor by New York Heart Association functional class in patients with CHF [38].

##### Health Status

Health status was assessed with the 36-item Short Form Health Survey (SF-36) [39]. The SF-36 is a self-report measure composed of eight multi-item scales: physical functioning, social functioning, vitality, role limitations (physical problems), role limitations (emotional problems), general mental health, bodily pain, and general health perception. The raw scores are transformed into scale scores ranging from 0 to 100, with a higher score indicative of a better health status (e.g., the absence of pain for the bodily pain scale). The SF-36 has good psychometric properties, with Cronbach’s *α* ranging from 0.78 to 0.93 for the separate subscales in a sample of 4,172 Dutch adults [39].

### Statistical Analyses

The chi-square test (Fisher’s exact test when appropriate) was used to compare nominal variables, whereas Student’s *t* test for independent samples or ANOVA (or the Mann–Whitney *U* test or the Kruskal–Wallis test, as appropriate) was used to compare continuous variables between groups. The relationship between two continuous variables was assessed with Pearson’s or Spearman’s rho correlation analyses, as appropriate. Multivariate logistic or linear regression analysis was performed, as appropriate, to investigate the relationship between type D personality and a history of anxiety or depressive disorder, anxiety or depressive symptoms prior to ICD implantation, and health status domains. A priori based on the literature on the associates of distress and health status in ICD patients, we chose to include sex, age, ICD indication, etiology, NYHA functional class, and LVEF in adjusted analysis. Given that the comorbidity of anxiety and depression is high, with a prevalence of 50–60 % [40], depressive symptoms were not included as a variable in the analysis of the relationship between type D personality and anxiety symptoms. Likewise, anxiety symptoms were not included as a variable in the analysis of the relationship between type D personality and depressive symptoms. Finally, for the same reason, a history of a depressive or anxiety disorder was not included as a variable in the analyses of the relationship between type D personality and anxiety and depressive symptoms. Variables were removed from the models, based on their statistical significance, to obtain parsimonious models. In case of multi colinearity, anticipated based on common sense and verified by *t* tests, chi-square tests, and Pearson’s correlation, various models were compared leaving out successive correlating variables. Model fit was then compared with either 2-log likelihood or *r*-squared, as appropriate. Effect modification by symptoms of anxiety and depression and a history of anxiety or depressive disorder in the relationship between type D personality and health status domains was anticipated. Therefore, interaction terms of type D personality and a history of anxiety or depressive disorder, and symptoms of anxiety and depression, were added to all final multivariate models with health status as outcome. If effect modification was present, stratified analyses by the effect modifier were performed. A *P* value of <0.05 was considered statistically significant. All analyses were performed with SPSS 15.01.

## Results

### Sample Characteristics

Between September 2007 and April 2010, 480 patients received an ICD at Medisch Spectrum Twente, Enschede, The Netherlands. Out of 347 eligible patients, 44 patients refused to participate. The remaining 303 patients were included in the TICS study.

Baseline characteristics for the total sample and stratified by type D personality are shown in Table 1. The prevalence of Type D personality was 21 %, depressive symptoms 15 %, and anxiety 24 %. A history of anxiety or depressive disorder was prevalent in 8 and 19 % of patients, respectively. Patients with a type D personality differed from non-type D patients on smoking, use of an aldosterone antagonist and use of a beta-blocker, etiology, age, and LVEF. On all other characteristics, type D patients did not differ significantly from non-type D patients.

**Table 1:** Baseline characteristics for the total TICS cohort and stratified by Type D personality

		Total (*N* = 278)	Type D (*N* = 59)	Non-type D (*N* = 219)	RR or mean difference	(95 % CI)	*P* value
Gender	Male	230 (83)	51 (86)	179 (82)	1		
	Female	48 (17)	8 (14)	40 (17)	0.74	(0.37; 1.50)	0.396
Age mean (SD)		62.6 (10.6)	60 (12.3)	63 (10.5)	2.99	(−0.35; 6.33)	0.079
Indication	Primary	196 (71)	43 (73)	153 (70)	1		
	Secondary	82 (30)	16 (27)	66 (30)	0.90	(0.57; 1.43)	0.652
Comorbidity	Diabetes	54 (19)	12 (20)	42 (19)	1.06	(0.60; 1.88)	0.841
	COPD	32 (12)	7 (12)	25 (11)	1.04	(0.47; 2.28)	0.924
	CVA	12 (4)	1 (2)	11 (5)	0.34	(0.04; 2.56)	0.368
	TIA	7 (3)	2 (3)	5 (2)	1.48	(0.43; 7.46)	0.360
Etiology	Ischemic	170 (61)	35 (60)	135 (62)	1		
	Non-ischemic	79 (28)	21 (36)	58 (27)	0.89	(0.71; 1.12)	0.292
	Other	29 (10)	3 (5)	26 (12)	0.53	(0.41; 0.68)	<0.001
Smoking		58 (21)	20 (34)	38 (17)	1.95	(1.24; 3.09)	0.005
Alcohol (use ≥1 glass/week)		175 (74)	34 (68)	141 (74)	0.91	(0.74; 1.12)	0.319
LVEF, mean (SD, *N* = 262)		30 (15)	26 (14)	30 (15)	−4.80	(−9.35; −0.24)	0.039
NYHA class	I	36 (13)	6 (10)	30 (14)	1		
	II	165 (62)	33 (56)	135 (57)	0.85	(0.38; 1.89)	0.680
	III	68 (25)	18 (31)	50 (23)	1.20	(0.90; 1.60)	0.259
Medication	Beta-blocker	229 (82)	52 (88)	177 (81)	1.07	(1.097; 1.22)	0.211
	Amiodarone	27 (10)	8 (14)	19 (9)	1.55	(0.72; 3.38)	0.266
	Diuretics	184 (67)	44 (75)	140 (64)	1.16	(0.97; 1.39)	0.135
	Aldosterone antagonist	90 (33)	28 (48)	62 (28)	1.67	(1.18; 2.35)	0.006
	ACE-I/AT-II antagonist	216 (78)	44 (75)	172 (79)	0.95	(0.81; 1.12)	0.516
Depressive symptoms (cutoff ≥8)		42 (16)	22 (39)	20 (10)	4.02	(2.36; 6.82)	<0.001
Anxiety symptoms (cutoff ≥8)		66 (24)	27 (47)	39 (19)	2.53	(1.70; 3.75)	<0.001
History of depressive disorder		53 (19)	26(44)	27 (12)	3.57	(2.27; 5.64)	<0.001
History of anxiety disorder		21 (8)	10(17)	11 (5)	3.37	(1.51; 7.56)	0.002
Health status, mean (SD)	Physical functioning	60 (28)	50 (28)	62 (28)	−11.6	(−19.85; −3.39)	0.006
	Social functioning	71 (29)	58 (30)	75 (27)	−17.11	(−25.32; −8.87)	<0.001
	Role limitations (physical)	45 (45)	33 (42)	47 (45)	−14.22	(−27.45; 0.99)	0.030
	Role limitations (emotional)	67 (41)	48 (45)	72 (39)	−24.18	(−37.48; −10.88)	0.001
	Mental health	74 (19)	59 (21)	78 (17)	−18.58	(−24.73; −12.44)	<0.001
	Vitality	57 (23)	43 (19)	60 (22)	−16.87	(−23.36; −10.38)	<0.001
	Bodily pain	75 (27)	61 (30)	78 (26)	−17.60	(−26.39; −8.81)	<0.001
	General health	52 (20)	39 (18)	55 (20)	−15.31	(−21.12; −9.50)	<0.001

Presented as *n* (%) unless otherwise indicated

*RR* relative risk, *CI* confidence interval, *COPD* chronic obstructive pulmonary disease, *CVA* cerebrovascular accident, *TIA* transient ischemic attack, *LVEF* left ventricular ejection fraction, *NYHA* New York Heart Association functional class

### Type D Personality in Relation to Anxiety and Depression

In univariate analyses, type D personality was significantly associated with a history of anxiety (relative risk (RR) = 3.37) and depressive disorder (RR = 3.57) but also with symptoms of depression (RR = 4.02) and anxiety (RR = 2.53) prior to ICD implantation. After correcting for the confounding factors: age, gender, ICD indication, and etiology, type D personality was associated with a history of depressive disorder (odds ratio (OR) 6.2, *p* < 0.001) and a history of anxiety disorder (OR 3.9, *p* = 0.004). Furthermore, type D personality was associated with symptoms of anxiety (OR 4.0, *p* < 0.001) and depression (OR 5.9, *p* < 0.001) prior to ICD implantation.

### Associates of Health Status Prior to ICD Implantation

As shown in Table 2, type D personality was independently associated with poorer health status in the domains vitality, mental health, bodily pain, and general health. Anxiety symptoms and NYHA functional class were associates of health status, influencing all eight SF-36 domains. Other independent associates of poor health status were depressive symptoms (on three domains) and history of anxiety disorder (on one domain). In none of the final adjusted models effect modification was present by gender or ICD indication.

**Table 2:** Associates of health status

Health status domain	Constant	Type D^a^ (*P* value)	Anxiety symptoms^b^ (*P* value)	Depressive symptoms^c^ (*P* value)	History of anxiety disorder^d^ (*P* value)	History of depressive disorder^d^ (*P* value)	NYHA class (*P* value)
Social functioning	91.4	−7.0 (0.11)	**−17.4** (<0.001)	**−13.2** (0.010)	**−6.6** (0.30)	−1.2 (0.80)	−7.3 (0.019)
Vitality	70.3	−9.2 (0.007)	−13.6 (<0.001)	−4.7 (0.24)	**−0.2** (0.97)	**−2.6** (0.47)	**−12.8** (<0.001)
Mental health	84.4	−11.5 (<0.001)	−17.9 (<0.001)	−2.7 (0.39)	**−4.1** (0.29)	**−3.0** (0.29)	−3.3 (0.082)
General health perception	41.2	**−6.2** (0.04)	**−7.7** (0.01)	**−9.6** (0.008)	**−2.7** (0.54)	**−4.9** (0.13)	−8.0 (<0.001)
Role limitation (emotional)	94.0	**−10.2** (0.11)	**−24.3** (<0.001)	**−14.8** (0.04)	**−15.6** (0.09)	**−0.7** (0.92)	−16.8 (<0.001)
Bodily pain	94.8	**−10.1** (0.02)	**−10.8** (0.01)	−8.5 (0.10)	**−16.5** (0.01)	**0.0** (0.99)	**−6.6** (0.04)
Role limitation (physical)	96.5	**−1.7** (0.81)	**−19.2** (0.005)	−13.5 (0.10)	**−2.9** (0.77)	**5.5** (0.46)	−29.8 (<0.001)
Physical functioning	109.0	**−4.3** (0.32)	**−9.1** (0.03)	**−8.6** (0.09)	**1.8** (0.77)	**−7.0** (0.13)	−16.3 (<0.001)

The negative coefficients represent the amount by which the health status values are lower for patients with either a type D personality, anxiety symptoms, depressive symptoms, a history of anxiety or depressive disorder, and a lower NYHA functional class score, compared to patients without these factors. For each health status domain, only the significant covariates are presented. All analyses were adjusted for age, gender, LVEF, and ICD indication; these were not significantly related to any of the domains

*NYHA class* New York Heart Association functional class

^a^Type D caseness, as assessed with the Type D Scale (DS14)

^b^Symptoms of anxiety, as indicated by the Hospital Anxiety and Depression Scale (HADS)

^c^Symptoms of depression, as indicated by the HADS, depression component: depressive symptoms present

^d^History of anxiety or depressive disorder, as measured with the Mini International Neuropsychiatric Interview (MINI)

## Discussion

A paucity of studies have examined the level of psychological morbidity in ICD patients prior to implantation, which might potentially be an important determinant of psychological adaptation problems post-implantation. To our knowledge, no study has investigated the association between history of anxiety and depressive disorders, as assessed with a clinical diagnostic interview and patient-reported health status at the time of implantation in ICD patients. In the current cohort of ICD patients enrolled in the TICS study, we found that patients with a type D personality were more likely to have a previous anxiety or depressive disorder and to experience higher levels of anxiety and depressive symptoms prior to implantation. Patients with a type D personality also reported poorer health status in several domains of the SF-36 compared with non-type D patients, although the associations between type D and various domains of health status were also influenced by anxiety symptoms, depressive symptoms, a previous anxiety or depressive disorder, and NYHA functional class.

In a previous study in ICD patients, Sears et al. found that a history of depressive disorder was a predictor of poor health status at 8 and 14 months post-implatation [18]. In our study, we found that a history of an anxiety disorder was only associated with one of the eight health status domains of the SF-36 (i.e., bodily pain), while a history of a depressive disorder was associated with none of the domains in adjusted analysis. The association between bodily pain and history of anxiety disorder was also found in other populations—for example in the elderly [41] and in patients with chronic pain [42].

In the TICS cohort, we found that NYHA functional class, anxiety symptoms, and type D personality were the most important associates of poor health status, with the strongest and most consistent association found between anxiety symptoms and health status. Intuitively, we would have expected that personality factors would exert a stronger influence on health status as compared to anxiety, as anxiety is a mood state that may fluctuate over time and situations, whereas type D personality is a stable personality trait [43]. A possible explanation for this finding could be that the questionnaires were administered just prior to implantation, with anxiety representing preimplantation anxiety with respect to the upcoming ICD implantation.

Our results confirm those of previous studies on type D personality in ICD patients, showing that a subset of ICD patients has a greater risk of experiencing anxiety and depression symptoms, especially those with a type D personality [19, 44]. Previous research in cardiac patients, including ICD patients, has also shown that the presence of type D personality is not only associated with anxiety or depression symptoms prior to ICD implantation but with negative health outcomes, including mortality and morbidity as well [16, 19]. In a cohort of cardiac surgery patients, it was found that pre-surgery affective disorders, affective phenotypes, and personality traits (i.e., negative affect) were associated with post-cardiac surgery morbidity outcomes [45]. Moreover, there are indications that the preimplantation psychological profile remains unchanged during 1 year follow-up [46]. Therefore, the preimplantation psychological profile (i.e., type D personality, history of depressive or anxiety disorder, and preimplantation depressive or anxiety symptoms) of the ICD patient seems to be a factor that should be carefully monitored, since it appears to be associated with an increased risk of worse prognosis and poorer health status in the longer term [14, 15, 20, 44, 47].

Given the results of the current study, future studies should include stable psychological factors, such as type D personality, and a history of anxiety or depressive disorder, as the premorbid psychological functioning of ICD patients is likely to be an important predictor of psychological morbidity post-implantation. In clinical practice, knowledge of the patient’s premorbid psychological status may help health care professionals in managing and caring for ICD patients more optimally. These patients could be identified prior to implantation using either the brief 14-item Type D scale—in particular given that type D personality is also associated with a history of both anxiety and depressive disorder—or a more extensive diagnostic interview if a psychologist is accessible via psychological services of the hospital or the MINI as used in the current study.

This study has some limitations. First, anxiety and depressive symptoms were determined by means of self-report rather than a clinical diagnostic interview. A self-report measure can be influenced by social desirability. We countered this limitation by emphasizing confidentiality of all responses. However, in the current study, we also had information on past diagnoses of anxiety and depressive disorder using a clinical diagnostic interview. Second, the number of patients with type D personality was somewhat lower than that found in previous studies [16, 19, 48]. This could have led to a reduced chance to identify relevant associations with type D personality. Nevertheless, we found significant associations between type D personality and distress symptoms and health status, which is concurrent with previous research on type D personality [19, 49]. Third, the number of participants in some subgroups was quite small, as was the number of women in this study, potentially limiting the statistical power. Fourth, we had no information on baseline characteristics for patients who refused to participate in the study. It is possible that their demographic and clinical profile differed from that of the study participants, possibly limiting the generalizability of the findings to the total sample. Finally, we had no information on the psychological status of patients who refused to participate. These patients might be more distressed and experience poorer health than patients included in analyses.

In conclusion, this study showed that prior to ICD implantation, type D personality is independently associated with anxiety and depressive symptoms. Also, we found that type D personality is independently associated with a history of anxiety or depressive disorder in ICD patients. Furthermore, we found that type D personality, anxiety and depressive symptoms, and history of anxiety and depressive disorders comprise vulnerability factors for experiencing poor health status prior to ICD implantation. Future results from the TICS cohort will be able to demonstrate whether patients with a type D personality and a previous anxiety or depressive disorder will continue to experience more distress and report poorer health status than patients without these psychological characteristics, once follow-up data are available. Follow-up of the TICS study cohort will show whether patients with these characteristics may be at higher risk of morbidity and mortality, including ventricular arrhythmias necessitating ICD therapy. If these findings are confirmed, these patients should be carefully monitored, since psychological functioning is also associated with adverse health outcomes in ICD patients, including the occurrence of potentially life-threatening ventricular arrhythmias and mortality [7, 50].
